# Effect of personalized care based on OPT model on perceived control and quality of life among patients with breast cancer

**DOI:** 10.3389/fpubh.2023.1149558

**Published:** 2023-04-17

**Authors:** Rabigul Rahman, Parida Mamat, Haiyan Wang, Mili Nurtai, Maynur Mahsut, Zulhumar Ahmat, Mukadas Siyit, Hongmei Shang, Xiaoyan Zhang

**Affiliations:** ^1^School of Nursing, Xinjiang Medical University, Urumqi, Xinjiang, China; ^2^Breast Surgical Department, Oncology Center, The First Affiliated Hospital of Xinjiang Medical University, Urumqi, China; ^3^School of Public Health, Xinjiang Medical University, Urumqi, Xinjiang, China; ^4^Nursing Supervision Center, The First Affiliated Hospital of Xinjiang Medical University, Urumqi, China; ^5^Intensive Care Unit (ICU), The First Affiliated Hospital of Xinjiang Medical University, Urumqi, China

**Keywords:** OPT model, personalized care, breast cancer, perceived control, quality of life

## Abstract

**Introduction:**

Patients with breast cancer (BC) after surgery are prone to negative physiological and psychosocial discomforts which cause the poor quality of life (QoL) among the patients. Therefore, how to improve the disease management ability of BC patients and to alleviate these cancer-related negative experience are particularly important. This study purpose to explore the potential effects of personalized care based on OPT model on the perceived control and the QoL among patients with BC, and to provide effective clinical nursing intervention for BC patients.

**Methods:**

In this study, nonsynchronous controlled experiments were carried out on patients with BC, and the patients were randomly allocated to the control (*n* = 40) and intervention (*n* = 40) groups. The patients in the control group were given routine care; while the patients in the intervention group were given personalized care based on OPT model. The perceived control ability and QoL of the two groups were measured before and after the intervention.

**Results:**

There were no significant differences in the total score of cancer experience and control efficacy of BC patients between the control group (61.15 ± 5.659, 41.80 ± 4.702) and the intervention group (60.58 ± 7.136, 42.15 ± 5.550) before intervention (*p* > 0.05). After the intervention, the total score of cancer experience in the intervention group (54.80 ± 8.519) was significantly lower than that in the control group (59.575 ± 7.331), with significant differences (*p* < 0.05). The total score of control efficacy in the intervention group (49.78 ± 6.466) was significantly higher than that in the control group (43.32 ± 6.219), with significant differences (*p* < 0.05). Compared with the control group, patients in intervention groups showed significant improvement in QoL after the intervention (*p* < 0.05).

**Conclusion:**

Personalized care based on OPT model plays a significant role in improving the level of perceived control and the QoL among patients with BC.

**Clinical Trial Registration:**www.chictr.org.cn, ChiCTR2300069476.

## Introduction

Breast cancer (BC) is the leading malignancy in women around the world with 2.3 million new cases per year (1). In recent years, the incidence of BC in China has been increasing year by year (2). Thus the treatment of BC is facing significant challenges. Breast cancer is characterized by four main different types (Luminal A, Luminal B, HER-2-positive and TNBC) based on their expressions in estrogen (ER), progesterone (PR), human epidermal growth factor receptor-2(HER-2) and Ki67. According to different types of BC patients, the treatment method is different (3). The main treatments of BC are surgery plus adjuvant treatment, such as chemotherapy, radiotherapy, endocrine therapy and hormone therapy (4, 5). With advances in cancer treatment and early disease detection, the survival rates of patients with BC have increased. The efficacy of BC treatment has improved over the years and now gradually becoming available in developing countries, whereas it is widely accessible in most developed nations (6). However, these treatments can also cause adverse effects, including pain, fatigue and sleep disorders (7–10). He (11) found that more than 84% of Chinese patients with BC who received chemotherapy experience these symptoms, such as fatigue, sleep disturbance, and depression. These symptoms can significantly exerts a negative impact on the course and effectiveness of the patient’s treatment, affecting cancer-related morbidity and mortality, as well as quality of life, especially in the period after surgery (12, 13).

Breasts are emphasized by the society as a symbol of femininity, motherhood and sexuality. Patients with BC after surgery are prone to negative psychology such as inferiority complex, anxiety, hopelessness, depression, reduced sexual attraction and suicide due to the absence of a body part (breast) and fear of death (9). Therefore, how to improve the disease management ability of BC patients and promote their adaptability to the disease is particularly important.

Perceived control is an individual’s subjective perception, feeling or belief in control (14, 15), as an individual’s ability to maintain or recover relatively stable psychological and physical functioning during or after exposure to significant stressful life events (16–18). Barez (19) suggest that perceived control could be used as an early predictor of psychological adjustment to illness. Low level of perceived control have been linked to a variety of symptoms: higher anxiety (20), greater panic disorder severity and greater obsessive–compulsive symptoms (21). High level of perceived control helps patients develop a positive attitude towards their health and cope with their illness, thus it is associated with enhancing the effectiveness of treatments and improving patients’ QoL (17). Perceived control is not a stable, and it is susceptible to change. The life-threatening nature of the cancer disease and the extensive treatment modalities with uncertain outcomes of the disease may challenge one’s perceived control (16). On the other hand, there are evidences that it is possible to increase the sense of control with appropriate guidance and intervention.

Many researchers have carried out a series of research work to improve the level of perceived control and the QoL of patients with BC, such as psychological education intervention (22–26), nursing self-care educational intervention (27) and Web/or telephone intervention (28–31), physical activities (11, 32–35), the appearance care (36), self-disclosure intervention (37), community based intervention (38). Although these projects have achieved satisfactory results, there are few comprehensive methods including postoperative physical, psychological and social rehabilitation.

OPT theoretical model (39): Outcome-Present state Test(OPT)model is a recurrent, nonlinear clinical reasoning model. It emphasizes that nurses must repeatedly compare the evaluation data of patients’ current state and expected outcome state, constantly reflect on the current situation and problems, outcomes and measures, so as to make the best individualized nursing intervention and serves (40, 41). This OPT model requires nurses to constantly reflect on the situation and problems, as well as the results of patients, in order to make the best personalized nursing decisions. The personalized care model (42–44) has a positive effect on the nurses’ caring ability, not only to help build great relationships between nurses and patients but also to enhance the patients’ satisfaction and level of perceived control, enable them to actively participate in healthy behaviors, and improve their QoL.

This study was conducted to develop and investigate the effects of the personalized nursing intervention on the perceived control and QoL of patients with BC. The novel personalized nursing interventions were developed based on OPT model which deeply implement the “patient-centered” as nursing service concept, break the conventional nursing inherent mode, emphasize the important role of patients’ needs in nursing service. In this study, the nursing model consists of specific interventions such as Supportive Care Intervention, Psychological Health Education, Chinese Medicine Foot Bath Combined with Massage and Progressive Muscle Relaxation Training. The BC patients were able to choose the appropriate nursing intervention under the guidance of the medical staff. We hypothesized that the nursing intervention model could significantly improve patients’ level of perceived control and QoL. This study provides a reference for clinical nursing of BC patients undergoing surgery and has significant application value.

## Methods

### Design

**Figure 1:**
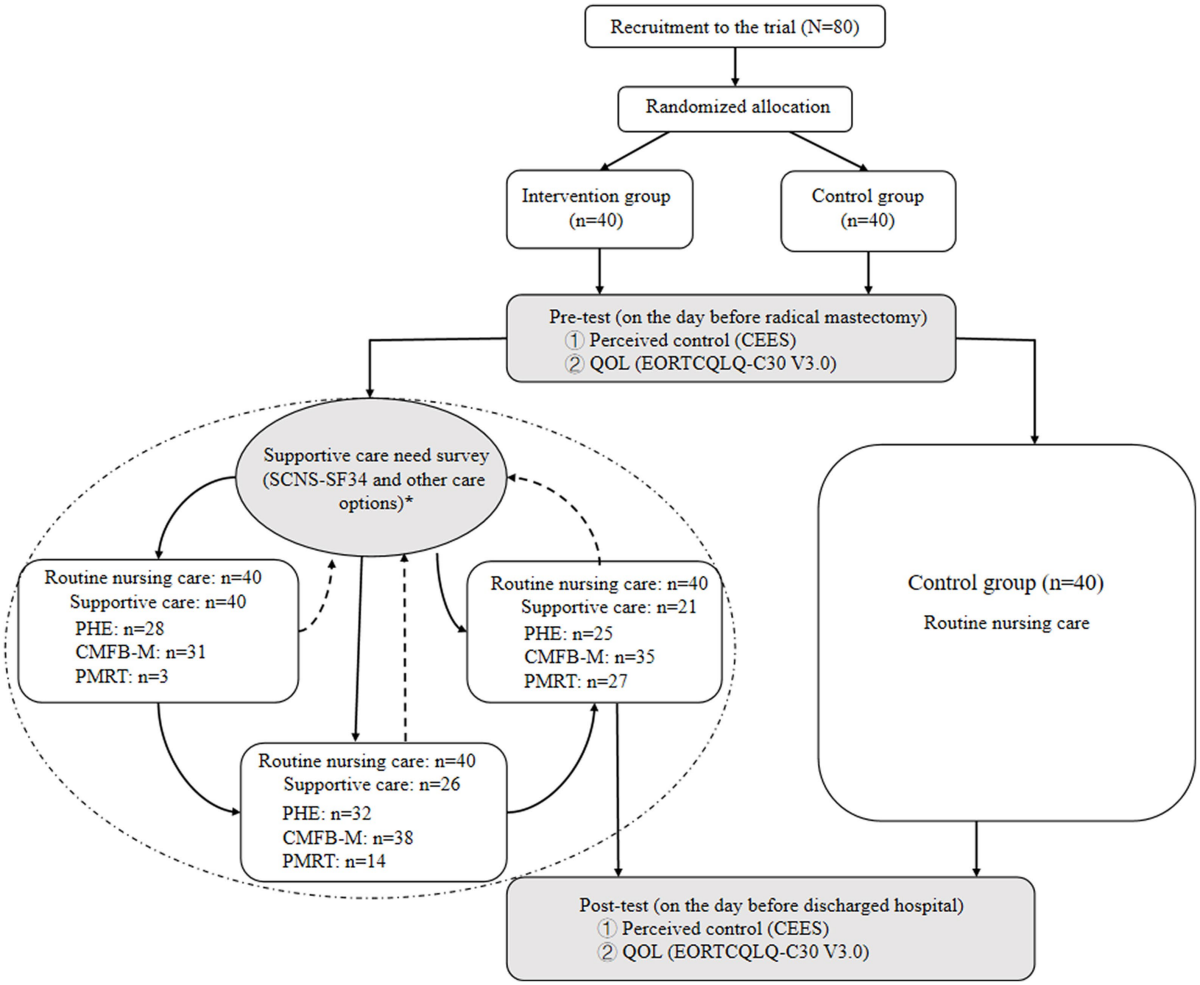
Flow chart of the personalized care program for patients with BC PHE: Psychological health education (afternoon 4 p.m.–6 p.m.Once every 3 days for 1 h) CMFB-M: Chinese medicine foot bath combined with massage (evening 21–22:30 for 30 min) PMRT: Progressive muscle relaxation training (early morning 8 a.m.–10 a.m for 30 min).

A randomized controlled trial design was used in the study. The protocol was reviewed and approved by the Human Research Ethics Committee of the First Affiliated Hospital of Xinjiang Medical University. The study flow diagram is illustrated in Figure 1.

### Setting

The trial was conducted in the breast surgical department of oncology center of the first affiliated hospital of Xinjiang Medical University, Urumqi, China.

### Participants

In this study, information about the study was given and informed consent was obtained from all the patients who agreed to participate in the study. Forty hospitalized patients with BC between December 2020 and May 2021 were selected as the control group; 40 patients with BC between June 2021 and December 2021 were selected as the intervention group. Patient inclusion criteria: (1) All patients were diagnosed as breast invasive ductal carcinoma by surgical pathology, and received modified radical mastectomy for the first time; (2) Combined with chemotherapy, radiotherapy, targeted drug therapy, endocrine therapy, etc. (3) Normal cognitive function and ability to cooperate with the study; (4) 30 ≤ age ≤ 82 years old; (5) Informed consent was obtained from all patients. Patient exclusion criteria: (1) Patients with severe heart, brain, liver and kidney function diseases; (2) Other cancers such as cervical cancer; (3) Infectious diseases; (4) Limb dysfunction; (5) Mental diseases; (6) Pregnant or lactating women.

### Statistical analysis and data analysis

The g-power 3.1 software was used for calculation, the t-test test method is selected, α is taken as 0.05, the effect value power is taken as 0.8, and the effect quantity D is 0.5. Using the bilateral test method, the sample size required for this study is 40 people in each group, and a total of 80 people.

The questionnaires were numbered in turn, and the data were recorded in Excel document after double check. After the data were checked again, they were imported into SPSS26.0 statistical software for data analysis. *p* < 0.05 means that the difference is statistically significant. (1) Patients’ general information, Supportive Care Need Survey Short-Form, (SCNS-SF34) (45), Cancer Experience and Efficacy Scale (CEES) (46), European Organization for Research and Treatment of Cancer Quality of Life Questionnaire Core 30 (EORTCQLQ-C30 V3.0) (47) scores were statistically described by frequency, percentage, c ± s. (2) Chi-square test or nonparametric Mann Whitney U-test were used to test the balance of general data of the two groups of subjects. (3) CEES, EORTCQLQ-C30 V3.0 were tested by normal distribution and ANOVA. (4) Repeated measurement analysis of variance was used to compare the intervention group and the control group in different intervention stages. The effect of two groups of nursing on CEES, EORTCQLQ-C30 V3.0 scores was discussed in detail.

### Research tools

(1) General information questionnaire: designed by researchers according to research needs, mainly including demographic and sociological data: age, education level, marital status, nationality, medical payment method, disease related data, etc.; (2) Personalized nursing needs assessment questionnaire for BC patients: the OPT model was taken as the theoretical basis, supported by the literature, conversation with BC patients, and self-made assessment questionnaire; (3) Supportive care need survey short form (SCNS-SF34): this scale is developed from cancer patients needs questionnaire (CPNQ), which can comprehensively evaluate the needs of tumor patients in all aspects; (4) Cancer experience and efficacy scale (CEES): It is divided into two parts: cancer experience and control efficacy, a total of 6 dimensions and 29 items. Cancer experience includes personal experience, socio-economic and emotional experience; control efficacy includes personal, group and medical efficacy. Grade 1–5 scoring method is adopted. The higher the score of cancer experience, the more negative experience the patient has, and the higher the score of control efficiency, the better the patient can cope with the disease. CEES is suitable for domestic cancer patients; (5) European Organization for Research and Treatment of Cancer questionnaire (EORTC-QLQ-C30): There are 15 fields in total, including 5 functional fields (body, role, cognition, emotion and social function), 3 symptom fields (fatigue, pain, nausea and vomiting), 1 general health status/quality of life field and 6 single items (each as a field), a total of 30 items. The scoring method of items 1–28 is divided into four levels from “none, a little, equivalent and extraordinary”; Items 29 and 30 are divided into 7 grades, which are 1–7 points according to the patient’s answer.

### Study design

Control group: the control group received routine nursing, the nursing content formulated by the Department, giving patients a comfortable environment, and routine admission education, diet guidance, medication guidance, activity guidance and discharge guidance during hospitalization.

Intervention group: patients in the intervention group were given routine care and personalized care based on OPT model during hospitalization. The personalized nursing services were selected by questionnaire survey among patients with BC, and the researchers formulated a personalized nursing intervention plan according to the assessment results of patients’ care needs. There are four nursing intervention including Supportive Care Intervention, Psychological Health Education (PHE), Chinese Medicine Foot Bath Bombined with Bassage (CMFB-M) and Progressive Muscle Relaxation Training (PMRT), etc. According to OPT theoretical model, a total of three sessions were provided in the program at lest during hospitalization. Each session, we provide personalized nursing services for patients according to their current nursing needs; Timely assess and understand patients’ satisfaction with nursing services, and constantly compare the current situation with the expected results to ensure the effectiveness of nursing services. The next session, patients are encouraged to choose the care method that is most suitable for them.

Supportive care intervention is according to the results of supportive care needs questionnaire (SCNS-SF34), provide targeted nursing services for patients.

PHE was delivered by a research team comprising three nurses and a psychologist. In this study, the psychoeducational intervention included psychoeducation on managing common symptoms in breast cancer patients and relaxation techniques. The general content of the psychoeducational intervention as follow: (1) Encourage personal introduction, expectations, and goals. Help patients find positive survival goals and build their confidence in life; (2) Encourage expression of feelings, thoughts, perceptions, anxieties and fears; (3) Provide accurate knowledge about BC and information about the common treatments; Pain control education; Provide nausea/vomiting and fatigue control education; Provide constipation and diarrhea control education; Sleep hygiene education; Coping improvement strategies; Problem-solving technique; Relaxation technique; Discuss communication skills.

CMFB-M was given to patients once a day for 30 min between 21:00 and 22:30 in the ward during hospitalization. The patients of this group were asked to sit on a chair with back support, then they immersed their feet in 40–45°C water of medicine prescription in electrical foot bath vessels for 20 min. The prescription mainly included Angelica sinensis, dried ginger and artemisia argyi leaves, etc. After that, patients receive massages focusing on the back, shoulder and arms for 10 min.

PMRT is the technique including continuous and systematic stretching and relaxing of the muscles until the whole body becomes relaxed (48). In this study, the patients were instructed how to contract and relax the 16 muscle groups on the third day after the surgery. Progressive muscle relaxation training was once a day, once in the early morning (8 a.m.10 a.m.) for 30 min per session until next survey. Progressive muscle relaxation training was conducted by the researchers in an orderly way and step by step according to the patients’ condition until the patients did the training freely and easily without uncomfortable feelings. Patients in the intervention group who chose this option attended and completed the training.

## Results

### Comparison of general data and demographic characteristics of BC patients

The general data of BC patients in different groups were compared among groups in terms of demographic characteristics such as age, nationality, residential area, education level and marital status. According to the test results, There was no significant difference in general data between the two groups (*p* > 0.05). It means that the research results between the intervention group and the control group were comparable after intervention. See Table 1 for details.

**Table 1:** General information of patients with breast cancer (*n* = 80).

		Groups				*t*/*χ*^2^	*p*
		Control group (*n* = 40)		Intervention group (*n* = 40)			
Mean age in years	(M ± SD)	54.55 ± 1.950		55.40 ± 1.530		−0.343	0.733
Ethnic Groups	Han	31	53.4%	27	46.6%	1.545	0.462
	Uygur	8	44.4%	10	55.6%		
	Other	1	25.0%	3	75.0%		
Education level	Middle school and below	13	50.0%	13	50.0%	0.080	0.994
	College and undergraduate	11	52.4%	10	47.6%		
	High school and technical school	15	48.4%	16	51.6%		
	Graduate and above	1	50.0%	1	50.0%		
Occupation	Cadres and retirees	23	53.5%	20	46.5%	2.796	0.424
	Individuals and others	3	75.0%	1	25.0%		
	Farmer	4	57.1%	3	42.9%		
	Unemployed	10	38.5%	16	61.5%		
Marital status	Divorced/widowed	2	40.0%	3	60.0%	1.588	0.452
	Single	1	100.0%	0	0.0%		
	Married	37	50.0%	37	50.0%		
Ways of detecting	physical examination	11	35.5%	20	64.5%	4.266	0.066
	Self-checking	29	59.2%	20	40.8%		
Mode of payment	Medical insurance for urban residents	10	58.8%	7	41.2%	4.340	0.227
	Other	4	26.7%	11	73.3%		
	New rural cooperative medical insurance	3	60.0%	2	40.0%		
	Employee medical insurance	23	53.5%	20	46.5%		
TNM stage	I	14	56.0%	11	44.0%	7.637	0.106
	II	20	51.3%	19	48.7%		
	III	3	75.0%	1	25.0%		
	IV	2	18.2%	9	81.8%		
	Unknown	1	100.0%	0	0.0%		
Household permanent residents	Living alone	4	40.0%	6	60.0%	0.463	0.793
	With spouse	34	51.5%	32	48.5%		
	With sons and daughters	2	50.0%	2	50.0%		
Family membership	Close	29	50.9%	28	49.1%	0.070	0.966
	Estranged	2	50.0%	2	50.0%		
	common relation	9	47.4%	10	52.6%		
Family history	No	35	49.3%	36	50.7%	0.125	0.723
	Yes	5	55.6%	4	44.4%		
Character	Introversion	20	48.8%	21	51.2%	0.269	0.874
	Extroversion	4	44.4%	5	55.6%		
	Moderate personality	16	53.3%	14	46.7%		

### Comparison of perceived control scores of different groups of BC patients before and after intervention

The results of this study showed that there were no significant differences in the total score of cancer experience and control efficacy of patients between the control group (61.15 ± 5.659, 41.80 ± 4.702) and the intervention group (60.58 ± 7.136, 42.15 ± 5.550) before treatment (*p* > 0.05). It means that the two groups of patients were in the same state before the intervention nursing. After the intervention, the total score of cancer experience in the intervention group (54.80 ± 8.519) was significantly lower than that in the control group (59.575 ± 7.331), with significant differences (*p* < 0.05). The total score of control efficacy in the intervention group (49.78 ± 6.466) was significantly higher than that in the control group (43.32 ± 6.219), with significant differences (*p* < 0.05). There were statistically significant differences in emotional experience, personal efficacy, collective efficacy, medical efficacy, total score of cancer experience and total score of control efficacy among different groups of BC patients (*p* < 0.05). See Table 2 for details.

**Table 2:** Comparison of perceived control scores of breast cancer patients in different groups before and after intervention (*n* = 80).

Variable	Groups	Before intervention	After intervention	Score difference
Personal strain	Control	14.98 ± 1.819	14.90 ± 2.530	−0.080 ± 1.966
	Intervention	14.80 ± 2.928	13.73 ± 3.210	−1.080 ± 1.760
	*t*/Z*	0.321	−1.154	−2.131
	*p*	0.749	0.249^*^	0.033^*^
Socioeconomic strain	Control	22.40 ± 2.968	21.63 ± 3.310	−0.780 ± 3.034
	Intervention	22.85 ± 2.732	20.15 ± 3.711	−2.700 ± 2.524
	*t*/Z*	−0.596	−1.964	−3.279
	*p*	0.551^*^	0.050^*^	0.001^*^
Emotional strain	Control	23.78 ± 2.597	23.05 ± 2.611	−0.730 ± 2.353
	Intervention	22.93 ± 3.238	20.93 ± 3.925	−2.000 ± 2.572
	*t*/Z*	−1.114	2.851	−2.305
	*p*	0.265^*^	0.006	0.021^*^
Personal efficacy	Control	15.13 ± 1.977	15.85 ± 2.587	−1.580 ± 6.126
	Intervention	14.83 ± 1.960	18.15 ± 2.833	−5.780 ± 5.031
	*t*/Z*	−0.443	−3.791	−3.538
	*p*	0.657^*^	<0.001	<0.001^*^
Collective efficacy	Control	16.38 ± 2.284	16.85 ± 3.034	0.730 ± 1.261
	Intervention	16.28 ± 2.900	19.23 ± 2.769	3.330 ± 2.740
	*t*/Z*	−0.48	−3.657	−5.452
	*p*	0.631^*^	<0.001	<0.001
Medical efficacy	Control	10.3 ± 1.6045	10.63 ± 2.072	0.480 ± 1.261
	Intervention	11.05 ± 2.309	12.40 ± 1.750	2.950 ± 3.137
	*t*/Z*	−1.589	−4.138	−4.629
	*p*	0.112^*^	<0.001	<0.001
Total score of cancer experience	Control	61.15 ± 5.659	59.575 ± 7.331	0.330 ± 1.347
	Intervention	60.58 ± 7.136	54.80 ± 8.519	1.350 ± 1.955
	*t*/Z*	−0.149	2.687	−2.73
	*p*值	0.881^*^	0.009	0.008
Total score of control efficiency	Control	41.80 ± 4.702	43.32 ± 6.219	1.525 ± 2.364
	Intervention	42.15 ± 5.550	49.78 ± 6.466	7.625 ± 6.146
	*t*/Z*	−0.212	−4.546	−5.858
	*p*	0.832^*^	<0.001	<0.001

The mark “*” refers to the non parametric *Z* test.

### Comparison of QoL scores of BC patients in different groups before and after intervention

Before the intervention, there was no significant difference in the scores of QoL, functional area and symptom area between the two groups, illustrating that the two groups of patients were in the same state, thus the data was comparable. There was no significant difference in the scores of QoL before (55.208 ± 12.184) and after (57.083 ± 13.549) the intervention in the control group (*p* > 0.05). There was significant difference in the scores of QoL before (54.583 ± 11.156) and after (61.458 ± 17.772) the intervention in the intervention group (*p* < 0.05). In the intervention group, the scores of the three dimensions of physical function (PF), role function (RF) and emotional function (EF) after the intervention were higher than those before the intervention, and the difference was statistically significant (*p* < 0.05). There were no statistically significant differences in the scores of other dimensions (*p* > 0.05). See Table 3 for details.

**Table 3:** Comparison of quality of life scores of breast cancer patients in different groups before and after intervention (*n* = 80).

Variable	Groups	Before intervention	After intervention	Score difference
Physical(PF)	Control	71.500 ± 10.780	72.167 ± 10.770	0.667 ± 8.241
	Intervention	71.167 ± 11.536	82.167 ± 9.354	11.000 ± 13.380
	*t*/Z*	−0.147	−4.005	−4.159
	*p*	0.883	<0.001	<0.001
Role (RF)	Control	71.250 ± 18.867	72.500 ± 17.110	1.250 ± 14.805
	Intervention	70.417 ± 18.677	84.583 ± 17.455	14.167 ± 24.329
	*t*/Z*	−0.189	−3.203	−2.868
	*p*	0.85	0.001	0.005
Emotional (EF)	Control	69.375 ± 13.789	70.208 ± 13.594	0.833 ± 5.908
	Intervention	68.542 ± 13.802	81.042 ± 11.162	12.500 ± 13.074
	*t*/Z*	−0.251	−3.623	−5.143
	*p*	0.802	<0.001	<0.001
Cognitive (*CF*)	Control	67.917 ± 20.460	69.583 ± 15.963	1.667 ± 17.622
	Intervention	68.750 ± 18.942	74.583 ± 18.867	5.833 ± 20.517
	*t*/Z*	−0.314	−1.375	−1.519
	*p*	0.753	0.169	0.012
Social (SF)	Control	65.417 ± 20.460	66.250 ± 20.840	0.833 ± 12.489
	Intervention	66.667 ± 21.014	71.250 ± 20.321	4.583 ± 24.456
	*t*/Z*	−0.305	−1.223	−0.864
	*p*	0.76	0.221	0.039
Dyspnea (DY)	Control	12.500 ± 18.002	12.500 ± 18.002	0.000 ± 13.074
	Intervention	14.167 ± 18.316	13.333 ± 22.393	−0.833 ± 26.675
	*t*/Z*	−0.444	−0.14	0.177
	*p*	0.657	0.889	0.046
Insomnia (SL)	Control	32.500 ± 24.445	30.833 ± 19.078	32.500 ± 24.445
	Intervention	31.667 ± 22.583	27.500 ± 24.907	31.667 ± 22.583
	*t*/Z*	−0.033	−1.104	−0.033
	*p*	0.973	0.27	0.023
Appetite loss (AP)	Control	27.500 ± 26.026	28.333 ± 25.654	0.833 ± 15.991
	Intervention	29.167 ± 24.093	20.833 ± 23.495	−8.333 ± 26.954
	*t*/Z*	−0.432	−1.335	1.85
	*p*	0.665	0.182	0.038
Nausea /Vomiting (NV)	Control	34.583 ± 20.806	32.083 ± 18.639	−2.500 ± 11.664
	Intervention	34.167 ± 21.334	28.333 ± 18.179	−5.833 ± 18.701
	*t*/Z*	−0.085	−0.9	0.057
	*p*	0.933	0.368	0.034
Constipation (CO)	Control	15.833 ± 16.858	13.333 ± 16.538	−2.500 ± 13.893
	Intervention	15.000 ± 16.794	16.667 ± 21.350	1.667 ± 21.284
	*t*/Z*	−0.223	−0.781	−0.882
	*p*	0.824	0.437	0.0378
Diarrhea (DI)	Control	5.000 ± 12.054	4.167 ± 11.164	−0.833 ± 9.206
	Intervention	5.833 ± 12.827	5.833 ± 12.827	0.000 ± 15.097
	*t*/Z*	−0.301	−0.622	−0.29
	*p*	0.763	0.534	0.052
Fatigue (FA)	Control	45.833 ± 16.148	46.389 ± 15.691	0.556 ± 10.049
	Intervention	45.556 ± 16.072	40.278 ± 23.562	−5.278 ± 17.248
	*t*/Z*	−0.088	1.365	1.848
	*p*	0.93	0.176	0.032
Pain (PA)	Control	33.333 ± 16.879	30.833 ± 16.256	−2.500 ± 12.259
	Intervention	32.917 ± 12.792	27.083 ± 22.229	−5.833 ± 24.620
	*t*/Z*	−0.092	0.861	0.767
	*p*	0.927	0.392	0.046
Financial difficulty (FI)	Control	35.833 ± 25.473	35.833 ± 25.473	0.000 ± 13.074
	Intervention	33.333 ± 26.149	39.167 ± 29.125	5.833 ± 19.810
	*t*/Z*	−0.536	−0.542	−1.554
	*p*	0.592	0.588	0.013
Quality of life (QL)	Control	55.208 ± 12.184	57.083 ± 13.549	1.875 ± 9.894
	Intervention	54.583 ± 11.156	61.458 ± 17.772	6.875 ± 16.000
	*t*/Z*	−0.173	−1.366	−1.681
	*p*	0.863	0.172	0.047

The mark “*” refers to the non parametric *Z* test.

## Discussion

BC and its treatments are lead to a variety of physiological and psychological problems of patients, thus reducing the adaptability of patients to cancer, resulting in increased negative experience and reduced QoL (11). So how to improve patients’ cancer management ability and to alleviate these cancer-related negative experience are particularly important. Perceived control (49) refers to that individuals believe they have sufficient ability to deal with external adverse events and are full of confidence in the expected results. The subjective perception, feelings or beliefs generated by individual control can affect disease treatment. Perceived control can appropriately alleviate the negative experience of patients, increase the ability to confront diseases and improve their coping efficiency. Cancer patients with low perceived control are prone to cope with cancer passively, manifested in insufficient self-care, passive acceptance of health education, reluctance to participate in medical communication (50). Therefore, effective interventions should be given in time to improve their perceived control.

This study take the OPT model as the structural framework to construct the nursing intervention for BC patients during hospitalization, and target the satisfaction of the humanistic care needs of BC patients as the outcome goal. During hospitalization, the patients in the control group only received routine nursing, and the patients in the intervention group received routine nursing and personalized care based on the OPT model. In this study, firstly, researchers used the Personalized Care Support Evaluation questionnaire and the Patient Supportive Care Needs questionnaire to understand the nursing needs of BC patients. Secondly, data analysis was performed on the collected data and central problems were identified, and personalized nursing interventions were formulated and implemented to help patients correct bad habits and behaviors. Finally, the effectiveness and feasibility of nursing interventions in improving the perceived control level and QoL of BC patients were evaluated.

The results of this study showed that there were no significant differences in the scores of the cancer experience and control efficacy of BC patients between the control group and the intervention group before treatment (*p* > 0.05), it means indicating that the two groups of patients were in the same state before nursing. After treatment, cancer experience score in the intervention group was significantly lower than those in the control group, with significant differences (*p* < 0.05). The less the score of cancer experience, the less the negative experience of patients. The score of the control efficacy in the intervention group was significantly higher than those in the control group, with significant differences (*p* < 0.05). The higher the score of control efficacy, the better the patient could deal with the disease. There were statistically significant differences in the perceived control ability of two groups in six aspects (*p* < 0.05): Emotional experience, Personal efficacy, Collective efficacy, Medical efficacy, total score of cancer experience, total score of control efficacy, see Table 3 for details. The results demonstrated that personalized care based on the OPT model can improve the perceived control ability of BC patients. Because, the Supportive Care Intervention and PHE in the personalized care model presented patients with disease knowledge and daily guidance comprehensively, encouraged patients to actively express their views, made efforts to solve patients’ nursing needs, guided them to adjust their nursing plans and treatment goals according to personal economic conditions, physical conditions and social support, strengthened humanistic care, and encouraged them to participate in the process of making nursing plans and correct cognitive biases. In addition, the CMFB-M and the PMRT in the personalized care model make them comfortable and relieve stress, making them more confident. Therefore, after the intervention, the cancer experience was reduced, the cancer control efficiency was enhanced, and the overall perceived control ability was improved.

Quality of life is a person’s feeling of his position in the culture and value system, and it is a reflection of the collective concepts of health, psychological state, independence level, social relations and so on (51). According to the previous study about psychosocial consequences of cancer therapy (24), QoL is considered to be one of the important clinical outcomes, most of disease outcomes were assessed by QoL. The current medical model believes that the QoL can better reflect the treatment and rehabilitation status of patients. Medical workers should not only pay attention to the survival number of patients, but also the QoL of them (52). Van Dijck and others found (5, 53) that the QoL of patients with BC was significantly lower than normal women. Therefore, how to take effective intervention to improve the QoL of BC patients after surgery and improve their mood and expectancy level are great significance. Nurses can use good nursing intervention to give patients sufficient psychological comfort, and increase their confidence to overcome the disease, and improve the patients’ QoL.

The results of this study showed that there was no significant difference in the scores of QoL, functional areas and symptom areas between the two groups before the nursing (*p* > 0.05), it means that the two groups of patients were in the same state. After the intervention, there was no significant difference in the scores of QoL in the control group (*p* > 0.05). However, scores of physical function (PF), role function (RF) and emotional function (EF) of patients in the intervention group were higher than those before the intervention, and the difference was statistically significant (*p* < 0.05). Although there was no statistically significant difference in the scores of other (fatigue, insomnia, nausea and vomiting, pain and loss of appetite) dimensions (*p* > 0.05). The main reason was that the medicine the patients use in chemotherapy stages which has a different effect on patients such as allergy, nausea, vomiting, diarrhea, hair loss, abnormal of liver and kidney function. The scores of physical function (PF), role function (RF) and emotional function (EF) are higher than those before the intervention. See Table 3 for details. Hence, humanistic care based on OPT model can improve the QoL of patients. The reason may be that the intervention plans were formulated by the results of questionnaire survey and motivational interview with patients during the research process, and humanistic care was carried out for the weak cognitive areas and nursing needs of patients. According to the OPT theoretical model (39), it is emphasized that to make the best individualized nursing intervention, the medical staff must repeatedly compare the evaluation data of patients’ current state and expected outcome state. Patients were encouraged to participate in the formulation of nursing plans, and they were guided to review their reactions about adverse symptoms and negative emotions during the treatment. The patients were organized to exchange nursing effects and share experiences. Thus, effective interventions that are tailored to the individual condition have been established by continuous improvement. This tailored intervention effectively improved the perceived control of BC patients. Perceived control as a psychological variable plays an extraordinary role in individual life, reflected in cognitive, emotional and behavioral functions (54). Barez (19) found that improving patients’ perceived control ability would significantly reduce the burden of symptoms and achieve better clinical outcomes. Aburuz (50) also pointed out that enhanced perceived control can reduce complications and improve outcomes. Therefore, how to effectively increase the control efficacy and regulate the cancer experience are the key to improve the QoL of BC patients. In this process, the control efficacy of the patients played a significant role compared to cancer experience in the perceived control, and the patients can actively participate in decision-making process of treatment, and have a sense of control over treatment and physical discomfort, to take a more positive attitude to deal with discomfort. Therefore, medical staff should take effective interventions such as psychoeducational counselling which are more important than delivering simple information to improve perceived control, then patients have a clear understanding of the disease and are more willing to believe that they can overcome the disease. In this model, we adopted four different nursing interventions, and patients selectively received nursing interventions according to their own conditions. As a result, the all dimensions of QoL such as emotional dimension, functional dimension and physiological dimension of the patients in the intervention group were improved after the intervention. The results illustrated that the personalized care based on OPT model significantly improved patients’ level of perceived control and QoL.

The current study has three limitations. First, the cross-sectional design of this study is difficult to infer a time series between perceived control and QoL of hospitalized BC patients, and the long-term impact of the perceived control on QoL remains unclear. Second, the relatively small number of participants in this study (each group, *N* = 40) may lead to a large final deviation, future studies should expand the sample size. Third, we only used perceived control(CEES) and EORTC QLQ-C30 measures, future studies should also take multiple symptom-related measures.

## Conclusion

In this study, the personalized care based on OPT model which was developed according to BC patients’ nursing needs and preferences significantly improved the level of perceived control and QoL among the patients with BC. In this model, we adopted four different nursing interventions, and patients selectively received nursing interventions according to their own conditions. There were significant differences in the total scores of cancer experience (60.58 ± 7.136, 54.80 ± 8.519, *p* < 0.05), control efficacy(42.15 ± 5.550, 49.78 ± 6.466, *p* < 0.05) and QoL (54.583 ± 11.156, 61.458 ± 17.772, *p* < 0.05) of BC patients in the intervention group before and after intervention. However, There were no significant differences in the total score of cancer experience (61.15 ± 5.659, 59.575 ± 7.331, *p* > 0.05), control efficacy(41.80 ± 4.702, 43.32 ± 6.219, *p* > 0.05) and QoL (55.208 ± 12.184, 57.083 ± 13.549, *p* > 0.05) of BC patients in the control group before and after nursing.

The results demonstrated the effectiveness and feasibility of the personalized nursing model. In the future research, implications of the study’s findings for health care providers, patients, and families would be valuable. The approach can be extended to the clinical nursing of other cancers, such as gastric cancer, lung cancer, and intestinal cancer. The study highlights that meeting the nursing needs of patients has to be taken as the central goal in clinical practice. The study suggest that health care professionals are encouraged to pay enough attention and understand the nursing needs of patients, help cancer patients effectively cope with the disease, reduce their cancer-related negative experience, and improve the patients’ quality of life. This study provided a reference for clinical nursing of BC patients undergoing surgery and has significant clinical practice value.

## Data availability statement

The raw data supporting the conclusions of this article will be made available by the authors, without undue reservation.

## Ethics statement

The studies involving human participants were reviewed and approved by the Human Research Ethics Committee of the First Affiliated Hospital of Xinjiang Medical University. The patients/participants provided their written informed consent to participate in this study.

## Author contributions

RR: conceptualization, methodology, formal analysis, investigation, writing—original draft, and project administration. HW: data collection, methodology, visualization, investigation, project administration, and supervision. MN: methodology, formal analysis, writing, statistical analysis and data analysis, and validation. MM: data collection, methodology, visualization, investigation, and supervision. ZA: conceptualization, methodology, investigation, writing, and review and editing. MS: methodology, formal analysis, review and editing, and supervision. HS and XZ: data collection, data curation, data interpretation, and investigation. All authors contributed to the article and approved the submitted version.

## Funding

This study was financially supported by Natural Science Foundation of Xinjiang Uygur Autonomous Region (no. 2020D01C168). We sincerely appreciate the support.

## Conflict of interest

The authors declare that the research was conducted in the absence of any commercial or financial relationships that could be construed as a potential conflict of interest.

## Publisher’s note

All claims expressed in this article are solely those of the authors and do not necessarily represent those of their affiliated organizations, or those of the publisher, the editors and the reviewers. Any product that may be evaluated in this article, or claim that may be made by its manufacturer, is not guaranteed or endorsed by the publisher.
